# Measles outbreak after 12 years without endemic transmission, Portugal, February to May 2017

**DOI:** 10.2807/1560-7917.ES.2017.22.23.30548

**Published:** 2017-06-08

**Authors:** Francisco George, João Valente, Gonçalo F Augusto, Andreia J Silva, Natália Pereira, Teresa Fernandes, Paula Palminha, Bárbara A Aguiar, António Martins, Estêvão Santos, Paula Valente, Etelvina Calé, Ana Leça, Paulo J Nogueira

**Affiliations:** 1 Directorate-General of Health, Lisbon, Portugal; 2 National Institute of Health Dr Ricardo Jorge, Lisbon, Portugal

**Keywords:** Portugal, viral infections, measles, measles-mumps-rubella (MMR) vaccine, outbreaks, surveillance, epidemiology

## Abstract

We report a measles outbreak in two Portuguese health regions (Algarve and Lisbon and the Tagus Valley) since February 2017, and which by 31 May resulted in 28 confirmed cases, of which 16 were unvaccinated. Thirteen cases were healthcare workers. One unvaccinated teenager died. Genotype B3 was identified in 14 cases from both regions. This outbreak occurs after 12 years without endemic measles transmission, and in a context of high measles vaccination coverage and immunity.

We describe a measles outbreak that started in February 2017, with 28 confirmed cases, as at 31 May. The investigation is ongoing and we present here preliminary findings and the implemented control measures. After the identification of the first measles case, a contingency plan at the Directorate-General of Health (Direção-Geral da Saúde, DGS) was implemented, following the Portuguese National Programme for Measles Elimination (Programa Nacional de Eliminação do Sarampo, PNES) [1]. This report is based on data extracted on 31 May from the National System for Epidemiological Surveillance (Sistema Nacional de Vigilância Epidemiológica, SINAVE), which is an integrated clinical and laboratory system of mandatory notification.

Vaccination against measles is included in the national immunisation programme (Programa Nacional de Vacinação*,* PNV) since 1974 and is available for free. This outbreak occurs after a 12-year period without endemic measles transmission in Portugal, which led the World Health Organization (WHO) Regional Office for Europe to certify measles as eliminated in the country in 2015 and 2016 [2]. In Portugal, measles is a mandatory notifiable disease since 1987 and, within the scope of the PNES [1], any suspected measles case notified is investigated thoroughly.

Immunity against measles is high in Portugal. The National Serological Survey conducted in 2001–02 showed a proportion of immune individuals above 93.4% in all age groups [3]. Preliminary results from the latest survey (2015–16), show that this trend of high immunity was maintained (data not shown).

## Case definition

The measles case definition used during this outbreak was based on the European Commission case definition [4]. Measles cases were defined as possible, probable or confirmed, depending on clinical, epidemiological and laboratory criteria.

A possible case was any person who met clinical criteria i.e. fever, maculopapular rash, and any of cough/coryza/conjunctivitis; a probable case was any person who met clinical criteria and had an epidemiological link to a confirmed case; a confirmed case was any possible case with laboratory evidence of infection with measles virus i.e. detection of viral RNA in a biological sample and/or a positive IgM result in serum, determined by the WHO-certified national reference laboratory for measles and rubella (National Institute of Health – Instituto Nacional de Saúde Doutor Ricardo Jorge, INSA) [5].

Following the WHO criteria [6], cases were discarded when clinical, epidemiological or laboratory criteria were not met, taking into account vaccination history and risk of measles infection in the community or abroad.

## Outbreak description

As at 31 May, 156 measles cases were notified in Portugal in 2017. In the outbreak reported here, 28 cases were confirmed, seven were classified as possible, three cases were under investigation and 117 cases were discarded. Additionally, one confirmed imported measles case was identified in the north health region, which was an isolated case with no epidemiological or genotypic links to the cases in the outbreak described here.

**Figure 1:**
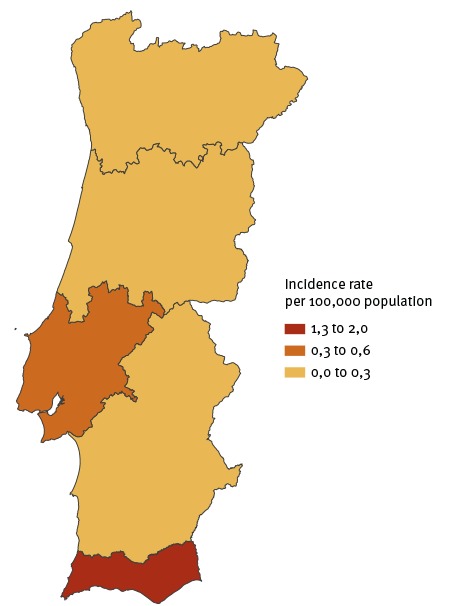
Incidence rate of confirmed measles cases per 100,000 population by health region, Portugal, 23 February–31 May 2017 (n = 28 confirmed cases)

All possible measles cases notified were investigated and control measures were promptly implemented in order to contain transmission. This report focuses on the description of confirmed cases in Algarve and Lisbon and the Tagus Valley (Lisboa e Vale do Tejo, LVT), two health regions which are not in close proximity (Figure 1). 

Map developed by GeoSaúde *(*http://www.geosaude.dgs.pt/).

Source: Direção-Geral da Saúde, Sistema Nacional de Vigilância Epidemiológica.

**Figure 2:**
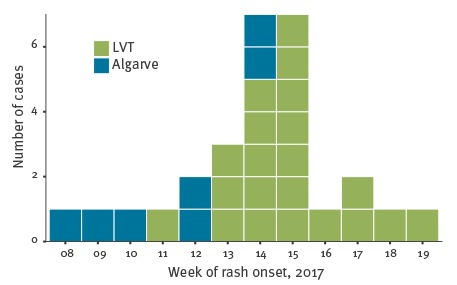
Confirmed measles cases by week of rash onset, Portugal, 23 February–31 May 2017 (n = 28)

The distribution of cases over time is described in the epidemic curve (Figure 2).

LVT: Lisboa e Vale do Tejo (Lisbon and the Tagus Valley).

Source: Direção-Geral da Saúde, Sistema Nacional de Vigilância Epidemiológica.

### Algarve health region

The first measles case was notified to health authorities on 30 March (rash onset on 21 March), and was from the Algarve health region, a touristic region with tourists mainly from other European countries. Local and regional public health teams immediately initiated epidemiological investigations that led to the retrospective identification of three additional cases, with prior disease onset. Overall, this transmission chain comprised seven confirmed cases (Figure 1, 2). The earliest case had rash onset on 23 February and the latest had rash onset on 8 April. No further confirmed cases were identified in Algarve. Five of these cases acquired measles in a healthcare setting, including two healthcare workers; five cases (4 infants aged under 1 year and 1 adult) were hospitalised. To date, no epidemiological link has been found between this cluster and other cases reported in Portugal or abroad.

### Lisbon and the Tagus Valley health region

The first case identified in the LVT health region was reported by a paediatrician from Cascais Hospital to the Regional Department of Public Health and the DGS on 6 April (rash onset on 30 March). Twenty-one cases were confirmed in LVT health region, with the earliest case having had rash onset on 17 March. This case was identified due to the epidemiological investigation of the first notified case in this region. The last confirmed case in the LVT region had developed rash on 13 May and no further measles cases were confirmed. The transmission chain in the LVT region is under investigation, with epidemiological links already identified between most cases. Eleven cases were healthcare workers. In the LVT region, seven cases were hospitalised, and one death has occurred in an unvaccinated teenager.

## Characteristics of cases

**Figure 3:**
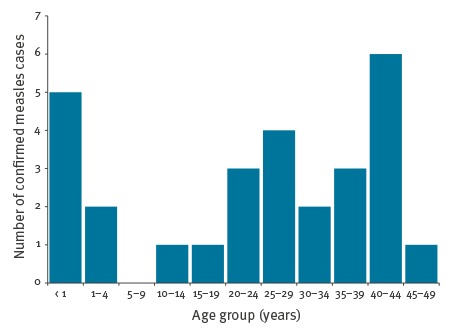
Confirmed measles cases by age group, Portugal, 23 February–31 May 2017 (n = 28)

Most confirmed measles cases (n = 19) occurred in adults (≥  18 years), two cases were adolescents (10–18 years), and seven cases occurred in children (< 10 years) (Figure 3).

Of note, 13 cases occurred in healthcare workers (Table). Of the 28 cases, 16 had not been previously vaccinated; while the remaining cases had documented evidence of one (4 cases) or two or more doses (8 cases, including 1 case vaccinated with 3 doses, the first one between 6 and 12 months of age) of a measles vaccine, either single or combined (Table).

**Table:** Characteristics of confirmed measles cases, Portugal, 23 February–31 May 2017 (n = 28)

Characteristic	Total (n = 28)
**Sex**	
Male	12
Female	16
**Occupation**	
Physicians	5
Nurses	5
Other healthcare workers	3
Other occupation / NA	15
**Vaccination status**	
2 or more doses	8
1 dose	4
Not vaccinated	16

NA: not applicable.

Among the unvaccinated cases, five were infants under 1 year of age and thus too young to be vaccinated, one case was 13 months old, two were adolescents, and the remaining eight cases were in adults (Figure 3). Thirteen cases were healthcare workers, of which three were unvaccinated.

## Laboratory investigation

Laboratory investigation was carried out by INSA. A variety of samples were used to confirm or discard a measles case: serum, oral fluids or throat swabs, or urine. Measles cases were confirmed by positive IgM, detection of measles nucleic acid or isolation of measles virus.

Genotype B3 was identified in 14 cases from Algarve and LVT health regions, identical to the genotype detected in other outbreaks in Europe in 2017 e.g. in Belgium, Italy and the United Kingdom [7,8]. Genotyping of the remaining 14 confirmed cases is still ongoing.

## Control measures

Following the increasing number of measles cases reported in several European countries in early 2017, DGS issued a warning to healthcare services, followed by recommendations and guidelines about diagnosis, early detection and response to measles cases, within the scope of the PNES [1].

In Portugal, when a suspected measles case is identified by health services, the physician is requested to fill in an electronic notification form at the SINAVE, and an email alert is simultaneously generated for local, regional and national public health authorities. Likewise, INSA also notifies DGS of all requests for measles tests, and notifies results electronically through SINAVE. Additionally, local public health units must inform immediately by phone and/or email regional and national public health authorities.

In this outbreak, all reported suspected measles cases were investigated and control measures were promptly implemented in order to contain transmission. While transmission could be prevented for imported case identified in the north health region, cases in Algarve and LVT had already generated transmission chains by the time they were reported.

Local public health teams undertook extensive contact tracing for all measles cases. Furthermore, surveillance and control measures included: immediate isolation of suspected cases, verification of immunisation status of close contacts, and administration of prophylactic immunoglobulin or measles-mumps-rubella (MMR) vaccine.

Epidemiological investigations and isolation of cases was complemented with broader public health measures, that included: (i) dissemination of key documents – posters about measles clinical picture, guidelines, epidemiological bulletins, and background materials for healthcare services – to support prevention and control measures [9-12]; (ii) creation of a specific section on measles on the DGS website [13], together with emails to inform healthcare professionals and schools; (iii) raising public awareness about the importance of vaccination through numerous reports in the media; (iv) enhancing vaccination, especially in children < 18 years and in healthcare workers, according to the PNV and PNES guidelines.

## Discussion

As at 31 May, 28 laboratory-confirmed measles cases were identified in Portugal in 2017. Transmission occurred in three different settings: household, community and healthcare services. Besides interviewing patients, hospital staff and family members, public health authorities had to liaise with airline companies and foreign public health authorities, as one confirmed case travelled abroad during the incubation period.

This measles outbreak in a country with high levels of reported vaccination coverage and immunity [3,14] represents a challenge for the Portuguese public health authorities. In contrast with many European countries, Portugal records high uptake of MMR vaccine. However, immunity gaps persist, with vulnerable population pockets [3,14,15]. We found a relatively large proportion of measles cases in vaccinated individuals and this has also been reported in other outbreaks [16-18]. This situation is expected in highly vaccinated communities and might be explained by the fact that MMR is not 100% effective, with around 7.5% and 5.0% non-respondents to the first and second doses, respectively [19].

Mass immunisation campaigns against measles took place in Portugal between 1973 and 1977. In 1974, the single measles vaccine was introduced in the PNV for children aged 12–15 months, and in 1987 the combined MMR. Since 1990, two doses of MMR vaccine are recommended for children. Since 2017, the recommended schedule is at 12 months and 5 years of age [20]. Two doses are also recommended for healthcare professionals, and one dose for adults born in or after 1970 without previous measles history [20]. Portugal has achieved sustained immunisation coverage against measles, above 95% for one and two doses in < 18-year-olds [21]. Measles immunity in older age groups was above 93% in 2002 [3].

Some delay between disease onset and notification in the early measles cases in Algarve and LVT could be explained by the fact that physicians might not have considered measles as the first diagnosis. This is very likely in a country without endemic measles transmission for more than a decade. After the first notifications, public health teams retrospectively investigated all potential measles cases, leading to the identification of four additional cases that had not been notified timely. The outbreak however made physicians more aware of the measles diagnosis and subsequent cases were notified timely.

The accumulation of measles-susceptible population pockets in a context of increasing number of outbreaks in European countries since 2016 [22,23] could have contributed to this outbreak in our country. Thus, possible links between these cases and other outbreaks in Europe are under investigation. The outbreak has increased national awareness and knowledge about measles diagnosis and epidemiological investigations, enhanced vaccination activities at the local level, and also motivated demand for vaccination from the general population and healthcare workers. The high and increasing number of notified possible measles cases being discarded in the course of this outbreak, increases confidence about its control. The last confirmed measles case in this outbreak was notified in the LVT health region on 14 May (rash onset on 13 May). No further confirmed cases linked to this case have been notified to date.
